# The incidence of venous thromboembolism in cervical cancer: a nationwide population-based study

**DOI:** 10.1186/1756-0500-5-316

**Published:** 2012-06-21

**Authors:** Shiang-Jiun Tsai, Ying-Xu Ruan, Ching-Chih Lee, Moon-Sing Lee, Wen-Yen Chiou, Hon-Yi Lin, Feng-Chun Hsu, Yu-Chieh Su, Shih-Kai Hung

**Affiliations:** 1 Department of Radiation Oncology, Buddhist Dalin Tzu Chi General Hospital, Chiayi, Taiwan; 2 Department of Chinese Medicine, Buddhist Dalin Tzu Chi General Hospital, Chiayi, Taiwan; 3 Department of Otolaryngology, Hematology, Buddhist Dalin Tzu Chi General Hospital, Chiayi, Taiwan; 4 Department of Oncology, Buddhist Dalin Tzu Chi General Hospital, Chiayi, Taiwan; 5 School of Medicine, Tzu Chi University, Hualien, Taiwan

## Abstract

**Background:**

Venous thromboembolism (VTE) is a life-threatening condition that occurs as a complication of cervical cancer. The aim of this study was to evaluate the incidence of VTE in cervical cancer patients during a 5-year follow-up.

**Methods:**

The study analyzed data deposited between 2003 and 2008 in the National Health Insurance Research Database (NHIRD), provided by the National Health Research Institutes in Taiwan. Totally, 1013 cervical cancer patients after treatment and 2026 appendectomy patients were eligible. The Kaplan-Meier method and the Cox proportional hazards model were used to assess the VTE risk.

**Results:**

The 5-year cumulative risk for VTE was significantly higher in the cervical cancer group than in the control group (3.3% *vs* 0.3%, *p* < 0.001). The hazard ratio for VTE was 10.14 times higher in the cervical cancer group than in the controls. The combined presence of more comorbidities was associated with a higher risk for VTE. Furthermore, cervical cancer patients without VTE had a significantly higher survival (75.3% *vs* 30.3%, *p* < 0.001).

**Conclusions:**

The cumulative risk of VTE was significantly higher in cervical cancer patients, and these patients also had lower survival rates. Strategies to reduce these risks need to be examined.

## Background

Cervical cancer remains the most important malignant disease in women, with an age-adjusted incidence of 26.2 per one hundred thousand people in Taiwan.[1] Venous thromboembolism (VTE) is a life-threatening condition in cervical cancer. Its incidence can be stratified by patient, tumor, or treatment. Reports of the VTE incidence in cervical cancer vary, and range from 0% to 34%.[2] For cancer-related VTE, the incidence of VTE is higher in the first few months after cancer is diagnosed, and subsequently decreases with time.[3,4] A literature review indicates that surgery, chemotherapy, or radiotherapy can be risk factors for VTE.[5] Radiation-induced vascular disease has been reported. Concurrent chemotherapy and radiotherapy is the standard treatment for patients with advanced cervical cancer, and it increases survival.[6] Although the number of long-term survivors has increased, data on the incidence of radiotherapy- or chemotherapy-induced VTE remains limited. Thus, the aim of this study was to evaluate the incidence of VTE in cervical cancer patients during a 5-year follow-up.

## Materials and methods

The study analyzed 2003–2008 data from the National Health Insurance Research Database (NHIRD), provided by the National Research Institutes in Taiwan. The NHIRD contains the medical benefit claims for 97% of the population from a registry of board-certified physicians and contracted medical facilities. The procedures we followed were in accordance with the ethical standards of the committee on human experimentation of our institution and with the Helsinki Declaration. This study was approved by the Institutional Review Board at Buddhist Dalin Tzu Chi General Hospital and approved number is B10001017.

The study included two cohorts. The principal diagnosis in the first cohort was cervical cancer, identified by the International Classification of Disease, Ninth Revision, Clinical Modification (ICD-9-CM) code 180. The different treatment modalities included surgery alone (S), radiotherapy alone (RT), surgery plus radiotherapy (SRT), surgery plus chemotherapy and radiotherapy (SCRT), surgery plus chemotherapy (SCT), chemotherapy and radiotherapy (CCRT), and chemotherapy alone (CT). Participants in the second cohort, who served as a control group, underwent appendectomy (ICD-OP code 47). Appendectomy patients were selected as a control group because of their similarity to the general population.[7,8]

Data on each patient were collected starting from the first hospitalization or outpatient visit in 2003. Totally, 1013 cervical cancer patients after treatment and 2026 appendectomy patients were eligible. Because of significant differences in mean age and comorbidities between the 2 groups, the control cohort criteria were further refined by randomly selecting 1013 matched female appendectomy patients at 1: 2 ratio based on age and comorbidities.

The primary dependent variable was venous thromboembolism (VTE; ICD-9-CM codes 415.11,415.19,451.11,451.19,451.81,453.40,453.41,453.42,4538–4539). In both groups, subjects who suffered a VTE and diagnosed before the index date were excluded from the data analysis. Patients were also excluded if distant metastases were diagnosed at initial.

Deaths recorded in the database were marked to calculate the vascular event-free survival, with cases censored if the patients died from non-vascular causes during follow–up. The independent variables were age, comorbidities, geographic region, urbanization level, and socioeconomic status. Comorbidities included hypertension, diabetes, coronary heart disease, and hyperlipidemia. There were four geographic regions (Northern, Central, Southern, and Eastern) and three urbanization levels (urban, suburban, and rural). This study also used enrollee category (EC) as a proxy measure for the socioeconomic status. All patients were categorized as EC1 (the highest socioeconomic status), EC2, EC3, or EC4 (the lowest socioeconomic status). These variables were associated with vascular disease.[9-11]

## Statistical analysis

The statistical software packages SAS (version 9.2; SAS Institute, Inc., Cary, NC, USA) and SPSS (version 17; SPSS Inc., Chicago, IL, USA) were used for data analysis. Inter-cohort differences in the frequency of variables were evaluated using the chi-square test. Cox regression model analysis was used to calculate the effects of VTE events on the case and control groups after adjusting for confounders. The risk factors included age, comorbidities, geographic region, urbanization level, and socioeconomic status. The vascular event-free survival was calculated using the Kaplan-Meier method. *P* < 0.05 was defined as statistically significant.

## Results

The distribution of demographic characteristics and comorbidities for the two cohorts is shown in Table 1. Compared to the control group (after matching), the case group had a high prevalence of hypertension, coronary heart disease, hyperlipidemia, and diabetes. There were 321, 162, 246, 69, 43, 36, and 624 patients in the RT, SRT, CCRT, SCRT, SCT, CT, and S groups, respectively. The 5-year cumulative risk of VTE in the RT, SRT, CCRT, SCRT, SCT, CT, and S groups was 3.0%, 3.8%, 3.0%, 3.0%, 6.5%, 11.0%, and 2.1%, respectively. If we excluded chemotherapy alone, there were no significant differences in the prevalence of vascular events between different treatment modalities. In addition, the 6 months, 1 year and 5 year cumulative risk of VTE in all case groups were 0.5%, 1.4% and 2.9%, respectively.

**Table 1:** Demographic characteristics and comorbidities of the cervical cancer and control groups

**Variable**	**Cervical cancer group (N = 1,013) No.(%)**	**Control group (N = 2,026) No.(%)**	***p***
Age, year			<0.001
≦44	366 (36.1)	641 (31.6)	
45–54	325 (32.1)	556 (27.4)	
55–64	155 (15.3)	351 (17.3)	
65–74	130 (12.8)	325 (16.0)	
≧75	37 (3.7)	153 (7.6)	
Hypertension			<0.001
Yes	256 (25.3)	395 (19.5)	
No	757 (74.7)	1631 (80.5)	
Diabetes			0.22
Yes	130 (12.8)	229 (11.3)	
No	883 (87.2)	1797 (88.7)	
Coronary heart disease			<0.001
Yes	92 (9.1)	90 (4.4)	
No	921 (90.9)	1936 (95.6)	
Hyperlipidemia			<0.001
Yes	36 (3.6)	30 (1.5)	
No	977 (96.4)	1996 (98.5)	
Geographic region			0.008
Northern	444 (43.8)	942 (46.5)	
Central	289 (28.5)	496 (24.5)	
Southern	262 (25.9)	518 (25.6)	
Eastern	18 (1.8)	70 (3.5)	
Urbanization level			0.06
Urban	268 (26.5)	618 (30.5)	
Suburban	464 (45.8)	870 (42.9)	
Rural	281 (27.7)	538 (26.6)	
EC			0.01
EC 1, 2	384 (37.0)	836 (41.3)	
EC 3	442 (43.6)	897 (44.3)	
EC 4	187 (18.5)	293 (14.5)	

**Figure 1:**
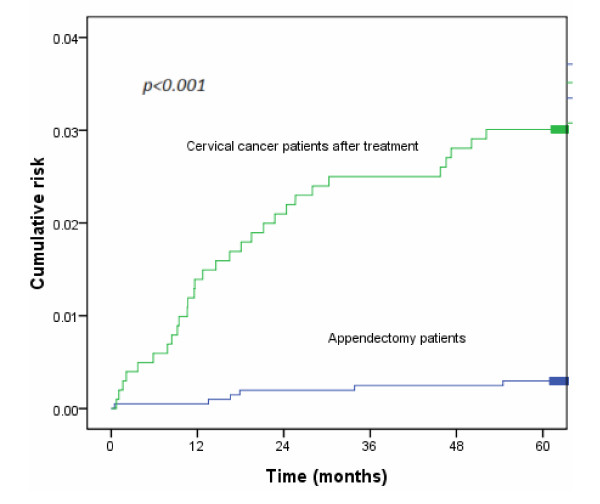
Figure reveals the cumulative risk of VTE in patients with cervical cancer and control patients.

**Figure 2:**
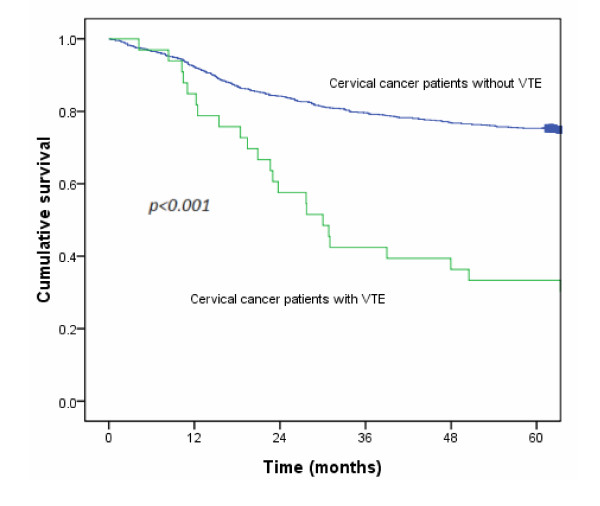
The survival of cervical cancer patients with VTE compared with survival in patients without VTE.

At the end of follow-up in 2008, a total of 39 patients had VTE, including 33 in the cervical cancer group and 6 in the control group. The median interval between treatment and the VTE event was 21.4 months. The average range for follow-up duration was 66.5 months. The 5-year cumulative risk of VTE was significantly higher for the cervical cancer group than for the controls (3.3% *vs* 0.3%, *p* < 0.001; Figure 1). Figure 2 shows survival in cervical cancer patients by comparing the vascular event of VTE. Cervical cancer patients without VTE had significantly higher survival (75.3% *vs* 30.3%, *p* < 0.001; Figure 2).

Unadjusted and adjusted hazard ratios for the association of VTE with cervical cancer after treatment and appendectomy are shown in Table 2. After adjustments for age and comorbidities, the hazard ratio for VTE during the 5-year follow-up was 10.14 times higher than among controls.

**Table 2:** Crude and adjusted hazard ratios for different vascular events in the 5-year follow-up period

		**Events (%)**	**Unadjusted HR (95%CI)**	***p***	**Adjusted HR (95%CI)**	***p***
VTE	Control group (N = 2026)	6 (0.3)	1		1	
	Cervix cancer group (N = 1013)	33 (3.3)	10.87 (4.54–25.99)	<0.001	10.14 (4.19–24.54)	<0.001

Adjusted for age, hypertension, diabetes, coronary heart disease, hyperlipidemia, geographic region, urbanization level, and enrollee category.

VTE indicates venous thromboembolism.

Five risk factors (age older than 55 years, hypertension, diabetes, coronary artery disease, and hyperlipidemia) were used to stratify the cervical cancer cohort into 2 groups: a low-risk group (n = 276; no risk factors), and high-risk group (n = 737; ≥1 risk factor). The 5-year cumulative risks of VTE in the stratified groups were 2.2% and 3.4%, respectively (Table 3).

**Table 3:** The annual VTE cumulative risk in different stratified groups

**N of Risk Factors**	**VTE events,N (%)**	**5-year VTE risk (%)**
0 (N = 276)	7 (2.5)	2.2
1 (N = 737)	26 (3.5)	3.4

Risk factors included age older than 55 years, hypertension, diabetes, coronary artery, disease, and hyperlipidemia.

## Discussion

Cervical cancer is an important health problem. Although its incidence is decreasing, it remains the most important cause of cancer death in women from Taiwan. VTE is a life-threatening condition in cervical cancer. It is important to record the incidence of VTE and design preventive strategies. For all cervical cancer patients, the 5-year cumulative risk of VTE was 3.3%. Our study reveals that cervical cancer patients have a higher cumulative risk of VTE as compared to the general population.

The incidence of VTE in cervical cancer can be stratified into patient, tumor, or treatment-related. However, it is difficult to separate these three categories. Few reports described the risk factors for patients with cervical-cancer-related VTE, which include age[12], comorbidities[13], immobilization[14], or inherited traits[15]. However, these reports were not recorded specifically in patients with cervical cancer.[2] For cancer-related VTE, Chew et al. reported the incidence of VTE from cancer diagnosis data. The VTE rate of uterus at one year was 1.6%.[16] Furthermore, the stage of the disease is defined by tumor extension and the extent to which it influences venous compression and stasis. Importantly, clinical findings reported an increased VTE risk in the advanced stages.[17] This means that the incidence of VTE correlates with the biological aggressiveness of the tumor.[18] Our study had similar reports, and cervical cancer patients without VTE experienced significantly higher survival rates.

Compared with the general population, cancer patients are often observed to have lower socioeconomic status.[19] This has subsequently been associated with a higher prevalence of comorbidities, such as diabetes mellitus, hypertension, or hyperlipidemia. These factors exacerbate vascular disease. In our study, five risk factors were used to stratify the cancer patients into low- and high-risk groups. The 5-year VTE incidence was lower in the low-risk group, 2.2%, than in the high-risk groups, 3.4%. Patients with more comorbidities had a higher risk of VTE. Therefore, interventions aimed at VTE prevention are extremely important. Complete surveys of modifiable risk factors and intensive lifestyle modification are recommended in patients with multiple comorbidities. In addition, some studies reported that low-molecular-weight heparin (LMWH) for thromboprophylaxis shows benefits for patient survival.[20] Further studies are recommended to determine the role in primary prevention of VTE.

Several limitations of this study should be mentioned. First, during data collection, the recording of VTE events in the NHIRD may have been inadvertently missed. In a multivariate analysis, the increased incidence of VTE was unrelated to the addition of platinum-based chemotherapy to radiotherapy. The relatively small size of the census populations and the relatively short follow-up period probably hindered the analysis. Second, the NHIRD is used primarily for administrative purposes and does not provide information on clinical characteristics, including staging, VTE severity, and biochemical data. Consequently, these pieces of data were not available for analysis in this study. Third, the NHIRD database does not have information on tobacco use, dietary habits, and body mass index, which may be additional risk factors for VTE. Forth, the vascular events in cervical cancer are low (only 33 events from 2003–2008), we preferred to use whole cervical cancer population to match control group. Although appendicitis is subject to Berkson’s bias, there are evidences that appendectomy patients could be selected as a control group because of their similarity to the general population.[7,8,21] Appendectomy patients are an acceptable comparator. Take together, given the magnitude and statistical significance of the effects observed in this study, these limitations are unlikely to alter our conclusion.

## Conclusion

In this cohort study, the cumulative risk of VTE was significantly higher in cervical cancer patients than in the general population. Cervical cancer patients with VTE had significantly lower survival rates. Strategies to reduce these risks need to be examined.

## Conflict of interest

The authors declare no conflicts of interest.

## Authors’ contributions

TSJ, RYX and HSK developed the ideas for these studies, performed much of the work, and drafted the manuscript. TSJ, CWY and HSK revised the manuscript. LCC, LMS, SYC, CWY and LHY designed the study, managed and interpreted the data. TSJ and HFC performed the statistical analysis. All authors read and approved the final manuscript.
